# Recent Advances in Nutritional Requirements and Metabolic Homeostasis Regulation of Animals Under Stress Conditions

**DOI:** 10.3390/ani15233412

**Published:** 2025-11-26

**Authors:** Xinhang Li, Xinjian Li, Zhenlong Wu, Kejun Wang, Ruimin Qiao, Xuelei Han, Xiuling Li, Feng Yang, Tong Yu, Tengfei Wang, Jun Bai

**Affiliations:** 1 College of Animal Science and Technology, Henan Agricultural University, Zhengzhou 450046, China; 19882957970@163.com (X.L.);; 2 Sanya Institute, Hainan Academy of Agricultural Science, Sanya 570228, China; 3 State Key Laboratory of Animal Nutrition, Department of Companion Animal Science, College of Animal Science and Technology, China Agricultural University, Beijing 100193, China

**Keywords:** animal stress, nutritional requirement, metabolism, mechanism

## Abstract

Animal stress is a complex physiological state characterized by a suite of non-specific responses (e.g., lethargy, anorexia, and impaired growth) to various internal or external threats, collectively known as stressors. It is the synthesis of non-specific responses produced by the body to various external or internal stimuli. Animals in livestock production are often stressed by breeding density, inappropriate temperature and humidity, harmful gases, noise and complicated immunization. Consequently, the nutritional requirements and underlying metabolic mechanisms in stressed animals are critical and growing research hotspots. Emerging evidence has shown that nutritional intervention can maintain animal health and performance under stresses. In addition, the regulation of metabolic pathways and targets under different stress states is also a potential way to alleviate the stress response of animals. With the rapid development of intensive farming and the aggravation of environmental changes, animals are facing increasing challenges such as heat stress, transportation stress, and pathogen infection. The resulting metabolic disorders and health problems seriously restrict the production efficiency of animals. This review aims to systematically analyze the variation in animal nutritional requirements under stress, clarify the molecular mechanism of metabolic imbalance, and summarize the regulatory targets and effects of nutritional intervention strategies, providing theoretical basis and technical support for solving stress-related problems in livestock production.

## Simple Summary

The current review provides a comprehensive classification of common stress types in animal production and detailed methodologies for establishing corresponding stress models. It thoroughly analyzes changes in nutritional requirements—particularly regarding proteins, amino acids, and energy—under stress conditions, and discusses dietary strategies to maintain animal performance. It integrates recent advances in molecular mechanisms, including neuroendocrine signaling, cytokine-mediated pathways, and oxidative stress responses, providing deeper insights into metabolic regulation under stress.

## 1. Introduction

Stress is an instinctive response of animals to external stimuli, mostly referring to a series of non-specific reactions produced by the body when it is stimulated by endogenous or exogenous stressors [1]. When the body is challenged by various unfavorable stressors from the inside or outside, homeostasis is disrupted and lost and stress occurs. In livestock production, common stressors include: environmental factors (e.g., heat, cold, humidity, concentration of toxic gases, radiation, trauma, etc.), disease factors (e.g., diarrhea, inflammation, bacterial infection, etc.), and feeding management (immunization, stocking density, transportation, etc.) [2,3,4]. However, regardless of the nature of the factors stimulating the organism, the stress response is essentially an adaptive and defensive physiological response displayed by the organism during the course of evolution to maintain normal life activities and ensure the recovery of the organism after injury or dysfunction [1,5].

Studies have shown that moderate stress can enhance the animals’ resistance to the external environment, thereby improving productivity and feed conversion rates [6]. Specifically, moderate stress can activate the stress response system of animals, allowing them to better utilize feed in the face of external challenges, which, in turn, enhances growth performance [7]. In addition, moderate stress may also enhance the growth rate of animals by promoting the secretion of growth hormone [8]. On the other hand, excessive stress can cause adverse reactions in animals such as loss of appetite, listlessness, reduced feed conversion rate, decreased production performance, weakened immunity, and even lead to diseases or death [9,10,11,12]. Current studies indicate that stressors such as environmental changes, nutritional deficiencies, and pathogen infections can lead to profound modifications in nutritional requirements and metabolic responses in livestock [13]. With the increasing concern for animal welfare and production efficiency, researchers have gradually recognized the importance of stress on the nutritional requirements of animals. Under stress conditions, the metabolic rate, energy expenditure and nutrient requirements of animals will change significantly. For example, heat stress can lead to decreased appetite, weakened digestion and absorption ability in animals, thereby affecting their nutritional status and growth performance [14]. Previous studies have also shown that transportation stress can lead to the release of large amounts of stress hormones in animals, such as cortisol, which can suppress appetite and decrease feed intake [15]. In addition, excessive cortisol not only directly affects the function of the digestive system but may also lead to metabolic disorders, thereby influencing the growth performance of animals [16]. Based on these findings, the physiological and biochemical mechanisms underlying these changes include alterations in hormonal signaling, immune response modulation, and metabolic shifts that promote energy homeostasis. These studies suggest the necessity for tailored nutritional strategies in livestock management, particularly under stress conditions. These strategies should focus on optimizing nutrient profiles to mitigate the negative effects of stressors on animal physiology and productivity. However, the research on the underlying mechanisms of nutrition metabolism in stressed animals is still not systematic and in depth, which makes the research on the nutritional requirements of stressed animals significantly limited. This review aims to synthesize the recent literature related to the construction of different stress animal models, the requirements and mitigation measures of different nutrients under stress, and the potential mechanisms of nutrient metabolism under different stress conditions, in order to establish a reasonable stress model and provide theoretical basis and potential improvement targets for the nutritional requirements of animals under stress.

## 2. The Types of Common Stress and the Construction of Stress Models in Animals

### 2.1. Immune Stress and Model Establishment

Immune stress refers to a non-specific immune activation state in which the immune system is repeatedly activated when the body is exposed to continuous antigenic stimulation (including both specific and non-specific), leading to characteristic clinical manifestations such as fever, anorexia, and immunosuppression [17,18]. In nutritional immunology, immune stress is regarded as one of the key factors that restrict animals from achieving optimal production performance and feed utilization efficiency. Evidence indicates that the inflammatory response can be regarded as a typical marker of immune stress, with its main features being elevated levels of cytokines such as interleukin-1 (IL-1), IL-2, IL-6, and tumor necrosis factor-α (TNF-α) [17,19,20]. At the same time, the production performance and immune function of animals will decline, and the contents of insulin-like growth factor (IGF-1) and growth hormone (GH) will also decrease accordingly [21,22]. To explore the direct impact of immune stress on animals and its potential mechanism of action, it is crucial to establish a successful immune stress model. The establishment of immune stress models includes methods such as lipopolysaccharide (LPS) induction, virus induction, and chemical/physical induction. Among them, LPS, as a commonly used stress inducer, is mostly administered by intraperitoneal injection in practical operations. Although the injection time and dose of LPS vary greatly in different studies, it can successfully induce an immune stress model. In pig production, porcine circovirus (PCV) infection poses a significant threat to piglets. Therefore, directly challenging livestock with pathogens (such as *Salmonella typhimurium*, *Actinobacillus pleuropneumoniae*, PCV-2, etc.) may be more relevant to production. In addition, restraint stress can also trigger an immune stress response in animals. Studies have shown that continuous restraint stress treatment of Bama miniature pigs for 18 days leads to an increase in oxidative stress levels in the intestine, resulting in intestinal mucosal damage, including impaired intestinal morphology and a reduction in the number of goblet cells and proliferating cell nuclear antigen-positive cells [23]. Table 1 describes the common methods for inducing immune stress and the applicable animals.

### 2.2. Oxidative Stress and Model Establishment

When livestock and poultry are exposed to stress stimuli, the imbalance of the redox system leads to excessive generation of highly reactive free radicals, which exceed the threshold of endogenous clearance capacity and induce peroxidation damage. Sohal et al. first proposed the concept of oxidative stress, which refers to the disorder of the redox system and the accumulation of free radical damage when the production rate of reactive oxygen species (ROS, including superoxide anion, hydrogen peroxide, and hydroxyl radicals, etc.) exceeds the clearance capacity of the organism, regardless of physiological or pathological conditions [34]. Under normal physiological conditions, ROS act as redox signaling molecules involved in metabolic regulation, and their levels are strictly regulated by the antioxidant defense system. However, excessive ROS under stress conditions can cause: (1) damage to biological membrane structure and cell function; (2) reduction in the thermal stability of nucleic acids and enzyme activity; (3) oxidation and denaturation of biological macromolecules such as lipids, proteins, and DNA, ultimately leading to metabolic disorders and increased susceptibility to diseases [35]. The clinical manifestations of this pathological process include behavioral abnormalities such as restlessness, vomiting, and anorexia, accompanied by increased levels of oxidative damage products such as malondialdehyde (MDA), protein carbonyl (PCO), and 8-hydroxydeoxyguanosine (8-OHdG) [36], reduced growth performance and meat quality [37], and damage to the intestinal barrier structure [38]. Common stressors used to establish oxidative stress models include hormones (hydrocortisone, corticosterone, dexamethasone, etc.), fatty acid substances (fish oil, soybean oil, coconut oil), diquat, dextran sulfate sodium (DSS), LPS, etc. [36,39,40,41]. Table 2 describes the common methods for constructing oxidative stress in animals, the applicable animals, and the core indicators.

### 2.3. Environmental Stress and Model Establishment

Environmental stress is particularly common in livestock production. It occurs when the environment itself or the tolerance of livestock to adverse conditions changes. The factors that affect the environment in which livestock live include: excessively high or low temperatures, excessively high or low humidity, transportation, noise, inappropriate lighting, excessive stocking density, and excessive levels of harmful gases [52,53,54]. In actual production, temperature stress is the main type of environmental stress, including cold and heat stress. Studies on heat stress are relatively more numerous, and its occurrence is due to a negative balance between the heat released by the organism into the environment and the heat produced by the organism (producing more than releasing). Heat stress reduces the feed intake of livestock, increases the consumption of nutritional metabolism in the body, and lowers the immune function, resulting in a decrease in the production and reproductive performance of livestock [55]. Low temperature is also one of the stressors. Different animals have different ranges and degrees of cold stress tolerance. Currently, there is no very precise definition for cold stress. It has been reported that low temperature increases environmental energy consumption and energy consumption within the animal’s body [56]. When the requirements for heat generation cannot be met, it also affects production performance. At the same time, cold stress can increase the levels of serum corticosterone, cortisol, and catecholamine hormones in the blood, significantly enhancing the activity of serum creatine kinase, and the effects on different animals still need further study [57,58]. Usually, the establishment of cold and heat stress models is achieved by controlling the environmental temperature of the livestock pens. Livestock are warm-blooded animals, and the external environment temperature at which chickens can maintain a normal body temperature is 16–32 °C [59]; newborn piglets are 27–29 °C, weaned piglets are 21–24 °C, growing and fattening pigs are 15–25 °C, sows giving birth and lactating are 16–18 °C [60], dairy cows are 10–15 °C [61]; sheep are 20–28 °C, and goats are 21–25 °C [62]. When the temperature maintained in the livestock housing environment is higher than the maximum suitable temperature for the organism, livestock will produce heat stress responses, establishing a heat stress model; when the temperature of the livestock breeding environment is lower than the minimum suitable temperature for the organism, or there is a sudden drop in temperature, or the environment is continuously below 4 °C, it will cause cold stress responses in livestock, establishing a cold stress model. Table 3 describes the common methods for constructing animal environmental stress models, the applicable animals, and the core indicators.

Overall, immune stress is primarily triggered by antigens and modeled via direct immunostimulants (e.g., LPS, viruses) or indirect stressors, leading to inflammation and performance decline. Oxidative stress, induced by chemicals, hormones, or diets, results from ROS overproduction and is marked by biomarkers like MDA and 8-OHdG. Environmental stress, mainly temperature-related, is modeled by controlling ambient conditions and manifests as physiological disruptions.

## 3. The Impact of Stress on Animal Nutritional Requirements

Stress is essentially a physiological response. In contemporary stress theory, the primary objective of the stress response is to activate all defense mechanisms and capabilities within the body. This mobilization aims to counteract the adverse effects of stressors and preserve the body’s homeostasis in extreme conditions. Additionally, it has been proposed that the concept of using nutrients to regulate the immune system function of animals, which is called immunonutrition (IMN) [75]. This concept applies to any situation where the inflammatory or immune response is regulated by changing nutrient supply. The activation of the immune system leads to a decrease in feed intake, weight gain, and protein deposition in livestock, thereby affecting production performance, changing nutritional requirements and patterns, especially for poultry that are sensitive to stress [76]. Under stress conditions, the catabolic metabolism of the body is significantly higher than the anabolic metabolism, manifested as negative nitrogen balance. This metabolic change is the result of neuroendocrine reactions. After stress occurs, the strengthening of catabolism inevitably leads to imbalance in the internal environment of the animal body, and at the same time, due to the large consumption of energy by tissues, the demand for vitamins and amino acids by the body increases sharply, and the deficiency of various nutrients will cause the body to quickly enter a state of exhaustion [77]. Studies have showed that the recovery from stress requires the body to have good physiological vitality, to quickly adjust the disordered physiological functions, and to accelerate the repair of damaged tissues [78,79,80]. Most stress regulation of nutritional metabolism mainly occurs through direct or indirect actions mediated by cytokines, and is reflected in the metabolic changes of proteins, fats, and carbohydrates.

### 3.1. Effects of Stress on Protein Nutritional Requirements of Animals

Studies have shown that low protein levels led to poor antioxidant stress resistance, while high protein levels resulted in better antioxidant stress resistance and superior meat quality [81,82,83]. Lee J et al. also observed a similar phenomenon in their study on fattening pigs under heat stress, where pigs fed low-protein diets showed decreased growth performance, nutrient digestibility, intestinal morphology, intestinal integrity, and serum antioxidant markers compared to those fed diets supplemented with lysine [84]. Therefore, when animals are under stress, supplementing them with a high-protein diet can effectively alleviate the impact of stress. However, when the external temperature changes, different physiological responses to heat and cold stress determine different dietary protein adjustments. Cold stress typically promotes increased feed consumption via hypothalamic appetite stimulation, often obviating the need for dietary protein escalation [85]. Conversely, the anorexia characteristic of heat stress compromises protein intake. Although increasing the dietary protein percentage can counter this deficit, caution is warranted due to the higher specific dynamic action (SDA) of protein, which augments metabolic heat production and may aggravate thermal load [86,87]. Therefore, it is recommended to balance the amino acids in the diet and formulate the diet based on the requirements of digestible amino acids to reduce the crude protein content while ensuring adequate amino acid intake, thereby preventing a decline in animal production performance. Morales et al. also demonstrated through their study on nutrient absorption in the intestines of pigs under heat stress that low-protein diets supplemented with amino acids improved intestinal absorption capacity compared to high-protein diets [88]. Furthermore, for weaned piglets, factors other than protein levels, such as palatability and the composition ratios of various feeds, also affect growth performance. However, most studies have indicated that there is no interaction between feed composition and immune stress on growth performance, but they can alleviate the growth retardation caused by stress [89,90,91].

### 3.2. Effects of Stress on Carbohydrate and Energy Requirements of Animals

Haisan et al. fed dairy cows with different levels of starch as energy sources from 28 days before parturition to 20 days after parturition [92]. The results showed that cows fed with higher levels of starch had higher milk production and lower levels of inflammatory factors in serum, indicating better resistance to immune stress. In addition, it has been reported that heifers were fed with different energy levels of diet for 45 days before parturient, and the high energy diet group showed stronger anti-immune stress and antioxidant capacity [93]. Adebowale et al. also found that weaned piglets require a higher energy feeding level when facing oxidative stress [94]. Moreover, with the deepening of research on nutritional metabolism and the deepening of understanding of the body system, the regulatory role of the immune system in nutritional metabolism has gradually received attention. Under different stress conditions, the digestion and absorption, synthesis and decomposition metabolism of nutrients in animals will change differently, and this process has dynamic characteristics. Therefore, it is difficult to determine the nutritional requirements of animals under different stress conditions through feeding experiments. Therefore, further research and discussion are still needed on the range of changes in nutritional requirements of animals under stress conditions.

## 4. Research on the Mechanism of Metabolic Regulation in Stressed Animals

### 4.1. Immune Stress and Metabolic Mechanisms in Animals

**Figure 1:**
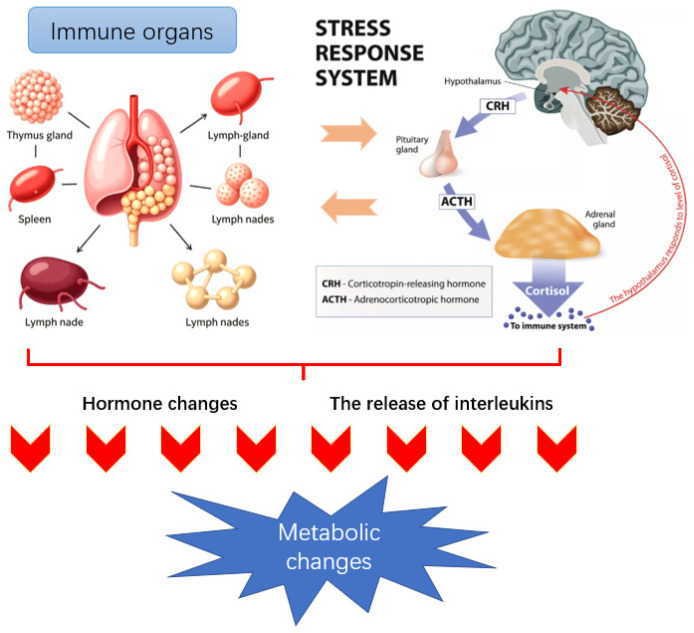
Immune stress and metabolic mechanisms in animals. Immune challenges (e.g., LPS or pathogens) activate immune cells to release pro-inflammatory cytokines (TNF-α, IL-1, IL-6), which stimulate the HPA axis. This leads to glucocorticoid release and endocrine alterations, suppressing anabolic hormones (e.g., IGF-1, GH) while promoting catabolism. Consequently, nutrient metabolism is reprogrammed away from growth and toward immune support, resulting in reduced performance and metabolic imbalance.

The immune system, as a sensing mechanism, can recognize the presence of antigens (such as bacteria, viruses, and exogenous proteins) within the body and transmit relevant information to other parts of the body, thereby triggering a series of changes at the behavioral, cellular, and metabolic levels [95]. The immune system-mediated growth or nutritional regulation mechanisms related to metabolism mainly include the following three aspects: (1) There is a direct connection between immune tissues (thymus, spleen, lymph nodes, etc.) and the central nervous system. Peripheral immune responses can activate the hypothalamic-pituitary-adrenal (HPA) axis in the central nervous system, thereby causing metabolic changes [96,97]. The specific process of HPA axis involvement in immune stress regulation is as follows: When immune stressors stimulate and activate the hypothalamus, the hypothalamus releases corticotropin-releasing factor (CRF); CRF acts on the pituitary gland, causing it to produce adrenocorticotropic hormone (ACTH); ACTH further activates the adrenal cortex, ultimately promoting the secretion of glucocorticoids and thereby causing metabolic changes. (2) There is a regulatory association between immune stress and the endocrine system. For example, the immune system can affect the metabolic process through hormones released by the pituitary gland. Specifically, immune stress reduces the secretion of growth hormone, increases plasma steroid hormone levels, promotes the secretion of catecholamines, and simultaneously lowers insulin-like growth factor levels. These changes alter the metabolic state of animals and their utilization and demand for nutrients [98]. (3) White blood cells release interleukins (including monokines and lymphokines, also known as cytokines). To resist the stimulation of external antigens, various immune active cells in the body secrete a series of cytokines to alter the body’s metabolism. Among them, the three monokines (TNF-α, IL-6, IL-1) produced by macrophages have a profound impact on the behavior, endocrine, and metabolism of animals during the growth process [22,99,100]. Figure 1 illustrates the process of immune stress and subsequent metabolic alterations in animals.

### 4.2. Oxidative Stress and Metabolic Mechanisms in Animals

**Figure 2:**
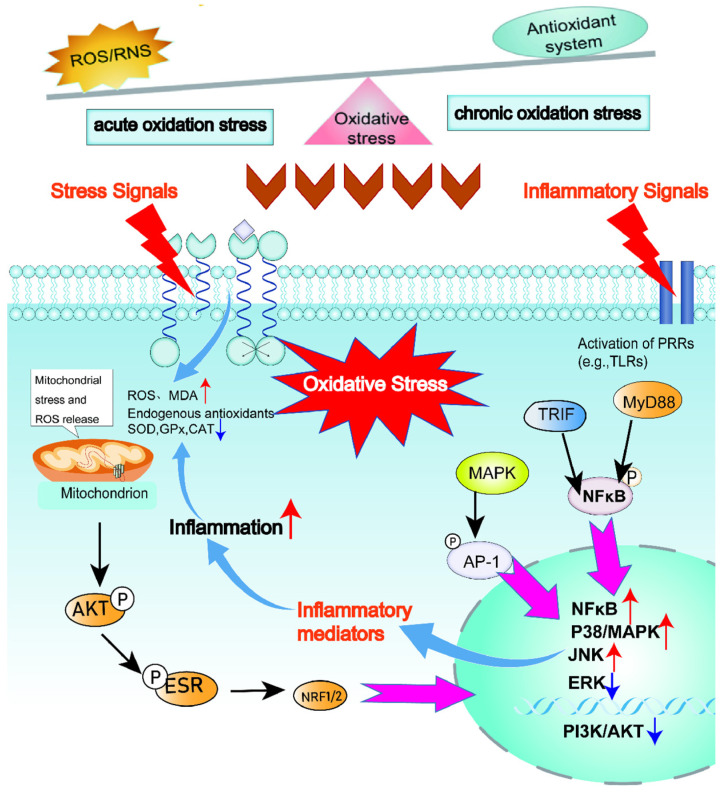
Oxidative stress and metabolic mechanisms in animals. Oxidative stress is an imbalance between the production of reactive oxygen species (ROS) and the body’s ability to neutralize them. It begins when ROS, primarily generated as metabolic byproducts during mitochondrial energy production, accumulate beyond the capacity of the antioxidant defense system, which includes enzymes like superoxide dismutase and catalase. This imbalance leads to widespread oxidative damage to cellular lipids, proteins, and DNA, disrupting normal function, promoting inflammation, and ultimately contributing to cellular dysfunction, aging, and the pathogenesis of various metabolic diseases.

When the content of reactive oxygen species (ROS) generated by the body’s metabolism exceeds its clearance rate, the redox balance within the cells is disrupted, thereby triggering oxidative stress responses. During this process, the large amount of free radicals produced cause damage to cells and tissues [101]. Under physiological conditions, redox homeostasis is maintained through intricate feedback mechanisms that regulate the generation and elimination of reactive oxygen species ROS and free radicals. This balance keeps ROS concentrations at specific, non-detrimental levels. Consequently, any basal oxidative damage that occurs is rapidly repaired, preventing its accumulation and ensuring cellular integrity. However, when the content of small molecule reducing substances responsible for eliminating ROS and free radicals decreases, or the activity of antioxidant enzymes is inhibited, the redox balance will be disrupted, thereby triggering oxidative stress responses [102,103]. Oxidative stress can be classified into three types: Firstly, acute oxidative stress, at which the rapidly increased ROS in the body can be fully cleared by the normal antioxidant system and restored to the normal level. The short-term increase in ROS does not immediately trigger oxidative stress but is accompanied by specific biochemical reactions, including the action of enzymes such as superoxide dismutase (SOD) and related antioxidants, to eliminate excess ROS and prevent the occurrence of oxidative stress [104,105]; Secondly, chronic oxidative stress, in this state, the high levels of ROS cannot be restored to normal levels quickly and require a slow reaction process, leading to the disruption of the body’s homeostasis [105,106]; Thirdly, the quasi-static level, in this state, the high levels of ROS in the body are regulated by reduction reactions and cannot return to the initial stable state but establish a new stable state of redox reactions. The excessive accumulation of reactive oxygen species (ROS) and reactive nitrogen species (RNS) not only triggers oxidative stress responses within the body but also causes mitochondrial dysfunction within cells [35,107,108]. Oxidative stress not only causes damage to cell membranes and proteins but also leads to base alterations. The activator protein-1 (AP-1) endonuclease and DNA glycosylase present in mitochondria and the nucleus can remove and repair the altered bases [109]. However, when the cumulative damage to DNA and proteins caused by oxidative stress continues to exceed the body’s own recovery and repair capabilities, it will trigger severe disease responses. To prevent oxidative stress from causing oxidative denaturation of bioactive substances, the body interrupts the synthesis reactions of a large number of substances during oxidative stress, thereby affecting the body’s material basis. The Nrf2/Keap1 pathway is the main approach for the body to respond to mild oxidative stress. This pathway regulates antioxidant-related genes, promotes the production of antioxidant enzymes and the synthesis of other antioxidants, thereby enhancing the body’s antioxidant response [110]. The immune regulatory system centered on AP-1, MAPK and NF-κB is the main regulatory system for the body in a severe oxidative stress state, by upregulating the expression of inflammatory genes and promoting the release of inflammatory factors [101,111]. The process of animal oxidative stress and metabolic mechanisms is shown in Figure 2.

### 4.3. Environmental Stress and Metabolic Mechanisms in Animals

**Figure 3:**
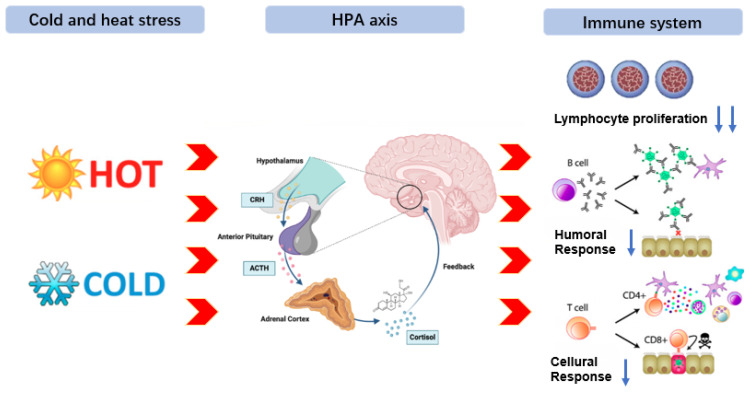
Environmental stress and metabolic mechanisms in animals. Environmental stressor (heat or cold) activates neuroendocrine axes (SAM, HPA, HPT), inducing release of cortisol, adrenaline, and thyroid hormones to promote adaptation. Concurrent HPA activation and glucocorticoid secretion suppress lymphocyte proliferation, impairing cellular and humoral immunity. This immune dysfunction increases infection susceptibility and diverts nutrients from growth and reproduction, resulting in substantial productivity losses.

Environmental stress refers to the pressure exerted by the external environment on the survival state of animals, which prompts the activation of response mechanisms in internal organs and tissues, thereby causing disruptions in normal physiological functions and leading to stress responses [112,113]. In practical production, animals are prone to varying degrees of physical damage due to the influence of the external environment during transportation. As the degree of stress intensifies, excessive energy consumption can suppress the immune system function. Moreover, due to individual differences, some animals can restore metabolic balance under environmental stress through self-regulation, but weaker groups may experience metabolic imbalance and physiological disorders when exposed to stress beyond their regulatory threshold [114]. Among environmental stresses in livestock production, heat and cold stress are the most studied. Heat stress is generally believed to reduce production and reproductive performance by directly lowering feed intake [115,116]. However, an increasing number of studies have shown that heat stress first reduces feed intake and nutrient absorption, thereby affecting the metabolic level of the body, especially promoting excessive production of free radicals and causing oxidative stress [117,118], ultimately leading to a decline in production and reproductive performance of livestock through cellular and mitochondrial oxidative damage [119,120,121]. Additionally, cold stress, as one of the stressors, can cause varying degrees of cold stress responses in animals exposed to it for a long or short period, which is also known as the “alarm response” [122,123]. Emerging evidence has shown that heat and cold stress affect the production and metabolism of animals, activating the three major nervous systems in the body, namely the sympathetic-adrenal medulla axis (SAM), the hypothalamic-pituitary-adrenal axis (HPA), and the hypothalamic-pituitary-thyroid axis (HPT), promoting the regulation of various hormones such as cortisol, thyroid hormones, and adrenaline to adapt to changes in the external environment as soon as possible [124]. Furthermore, such stressors can dysregulate immune responses, leading to a state of immunosuppression or excessive inflammation. This impaired immune competence not only increases vulnerability to infections but also diverts energy and nutrients away from growth, reproduction, and other production traits, resulting in significant economic losses [125,126]. The core pathway of immune suppression is: Environmental stressor → HPA axis → promoting the secretion of glucocorticoids → inhibiting lymphocyte proliferation → reduction in cellular and humoral immune functions [127]. The process of environmental stress and its mechanism in animals is shown in Figure 3.

## 5. Conclusions

In conclusion, stress represents a complex physiological response that significantly disrupts homeostasis and alters nutrient metabolism in animals, ultimately impacting health, welfare, and production efficiency. This review has summarized the common types of stress encountered in livestock production, namely immune, oxidative, and environmental stress, as well as methods for establishing representative animal models to study these conditions. A key consensus is that stress invariably increases the animals’ requirements for specific nutrients, including proteins/amino acids and energy-yielding components, while simultaneously impairing intake, digestion, and absorption efficiency. The underlying mechanisms involve intricate interactions between neuroendocrine pathways (e.g., HPA axis activation), immune responses (e.g., cytokine release), and redox signaling, which collectively reprogram metabolic priorities toward maintenance and defense at the expense of growth and productivity.

Despite advances in stress biology, current understanding of the precise alterations in nutritional metabolism under stress remains fragmented. The future research frontier will focus on integrating modern molecular nutrition, genomics, proteomics, and metabolomics, among other multidisciplinary approaches, to systematically analyze the macroscopic to microscopic changes in animal nutritional metabolism and absorption and utilization mechanisms under different stress conditions. Ultimately, integrating insights will be crucial for formulating targeted dietary interventions that enhance resilience, maintain productivity, and improve overall animal welfare in the face of unavoidable stress challenges.

## Figures and Tables

**Table 1:** Induction methods for immune stress in animals.

Category	Methods	Operation Points	Target Animals	References
Direct Immune Activators	1. Lipopolysaccharide (LPS) Injection	- Dose: Mice/Rats: 0.1–5 mg/kg Pigs: 10–100 μg/kg Chickens: 1–5 mg/kg Calves: 0.5–2 μg/kg - Peak Effect Time: 1–4 h	Mice, rats, pigs, chickens, calves, etc.	Zhou J [24], Pi CC [25], Agustinho BC [26]
	2. Yeast Polysaccharide Injection	- Route: Intraperitoneal injection - Dose: 10–100 mg/kg (mice) - Peak Effect Time: Several hours to 24 h	Rodents, poultry	Rahman MA [27]
	3. Viruses	- Route: Nasal drop/Tracheal injection - Dose: 10^3^–10^6^ EID_50_ (e.g., avian influenza virus); 10^4^–10^5^ TCID_50_ (PVC-2) - Monitoring: Viral load + inflammatory factors	Poultry, pigs	Chen S [28]
	4. Bacteria (e.g., *Escherichia coli*)	- Route: Intraperitoneal injection (10^7^–10^9^ CFU/mL) - Monitoring: Intestinal infection + inflammatory factors	Pigs, mice, etc.	Wang Q [29]
	5. Other PAMPs	- Polyinosinic-polycytidylic acid (activates TLR3), CpG DNA (TLR9), Peptidoglycan (TLR2)	Specific receptor mechanism research	Dietrich L [30]
Indirect Stressors	1. Management Stress	- Transportation, mixing/re-grouping, high-density housing, high/low temperature environment	Pigs, poultry, cattle, rodents	Langendijk PL [31]
	2. Physiological Stress	- Weaning (mother-offspring separation + nutritional change), restricted feeding, intense exercise	Piglets, calves, etc.	Wang L [32]
	3. Psychological Stress	- Restraint stress, social defeat stress	Rodents (rarely used in farm animals)	Nemeth M [33]

**Table 2:** Construction methods for oxidative stress in animals.

Stress Source Type	Representative Substance	Construction Method	Applicable Animals and Medium of Action	Core Oxidative Damage Indicators	References
Hormones	Dexamethasone	- Route: Intraperitoneal injection/Drinking water addition - Dosage: Mice/Rats: 1–10 mg/kg/d (injection) Chickens: 2–5 mg/kg/d (injection) - Duration: 3–7 days	Mice, Rats, Chickens	- Plasma MDA ↑, SOD ↓ - Muscle/Liver GSH consumption - Mitochondrial ROS generation ↑	Ulla A [42], Ambwani S [43]
	Corticosterone	- Route: Subcutaneous implant of sustained-release tablets - Dose: 10–40 mg/kg (sustained-release for 21 days) - Simulate chronic stress	Rats, Mice - Medium: FKBP51 HPA axis etc.	- Lipid peroxidation in hippocampus/prefrontal cortex ↑ - CAT activity in brain tissue ↓	Yuan T [44], Jafari Z [45]
Fatty acids	Fish oil (High polyunsaturated fatty acids)	- Feed addition: 5–10% as a substitute for oil - Period: 4–8 weeks - Induction mechanism: n-3 PUFA auto-oxidation	Rats, Carps, Broilers - Medium: PI3K-AKT-mTOR etc.	- Liver TBARS ↑, Carbonylated proteins ↑ - Antioxidant enzymes (GPx, GST) compensatory increase	Yi J [46]
	Soybean oil/Coconut oil (high in n-6 or saturated fatty acids)	- Feed addition: 10–20% as a substitute for base oil - Period: 6–12 weeks	Pigs, Mice, Laying hens - Medium: KEAP1- NFE2L2 pathway etc.	- Plasma 8-iso-PGF2α ↑ (lipid peroxidation marker) - Liver Nrf2 pathway activation	Lee DH [47]
Chemical inducers	Diquat	- Pathway: Intraperitoneal injection - Dose: Mice: 10–25 mg/kg Piglets: 8–12 mg/kg - Acute model: 6–24 h	Mice, Pigs, Zebrafish - Medium: NF-κB/mTOR etc.	- Systemic oxidative burst (SOD ↓, GSH ↓) - Multiple organs (Liver/Kidney/Gut) MDA ↑ - DNA oxidative damage (8-OHdG ↑)	Park A [48]
	Dextran sulfate sodium (DSS)	- Pathway: Oral drinking - Concentration: 2–5% (*w*/*v*) - Period: 7 days (Acute enteritis)	Mice, Rats - Medium: TNF-α, PD-1 etc.	- Colonic MPO activity ↑ (Neutrophil infiltration) - Intestinal mucosa SOD/CAT activity ↓ - Colonic tissue NOX2 expression ↑	Wang L [49]
	Lipopolysaccharide (LPS)	- Pathway: Intraperitoneal/Venous injection–Dose: Mice: 5 mg/kg Piglets: 100 μg/kg - Time: 4–24 h	Mice, Pigs, Poultry, etc. - Medium: ERK5 etc.	- Mitochondrial ROS release ↑ - Plasma antioxidant capacity (T-AOC) ↓ - Inflammation-oxidation interaction (iNOS induction → NO ↑)	Zhou Y [50], Cilenti F [51]

↑: Increase in expression level, ↓: Decrease in expression level.

**Table 3:** Construction methods for transportation stress in animals.

Model Name	Construction Method	Applicable Animals	Core Stress Responses	Operation Cycle	References
High Temperature Heat Stress	Constant temperature chamber/climate chamber: 35~40 °C, relative humidity 60~70% - Poultry: 38 °C for 72 h (acute) - Mammals: Day-night temperature difference cycle (32 °C day/28 °C night)	Chicken, pig, dairy cow, mice	- Respiratory rate ↑ - Plasma cortisol ↑ - HSP70 expression ↑ - Feed intake (>30%) ↓	3 days to 8 weeks	Silva PS [63], Koch F [64]
Low Temperature Cold Stress	- Constant temperature chamber: 4~10 °C (acute)/−5~0 °C (extreme) - Aquatic animals: Sudden drop in water temperature (e.g., 28 °C → 15 °C) - Mammals: Standing on ice (15 min/time × 3 times/day)	Zebrafish, mice, broiler chicken	- Activation of brown adipose tissue - UCP1 protein ↑ - Blood glucose first rises then drops - Atrophy of intestinal villi	24 h to 4 weeks	Zhang Y [64], Bornstein MR [65]
High Altitude Hypoxia	- Low-pressure chamber simulation: 5500 m (oxygen partial pressure ≈ 11.4%) - Combined operation: 30 min swimming in 22 °C cold water before entering the chamber - Continuous exposure: >72 h	Rat, mice, yak	- Brain tissue SOD ↓/MDA ↑ - Pulmonary artery pressure ↑ - Compensatory increase in red blood cells (HCT ↑)	3 to 21 days	Xue Y [66], Yang S [67]
Noise Stress	- White noise generator: 80~100 dB - Rhythm: Random intervals (to prevent adaptation) - Nighttime intensification: Silent during the light period, burst during the dark period (simulating sudden noise)	Rat, laying hen, pig	- Serum COR/ACTH ↑ - Apoptosis of hippocampal neurons ↑ - Egg production rate (in poultry) ↓	7 to 28 days	Lee J [68]
Disrupted Light Rhythm	- Inversion of day and night: 12 h light/12 h dark reversed - Continuous light: 24 h 200 lux - Flicker stimulation: 1 Hz flashing (5 min/h)	Mice, fruit flies, egg-laying ducks	- Disrupted melatonin secretion - Disordered clock genes (Bmal1, Per2) - Ovarian weight (in poultry) ↓	2 to 8 weeks	Ma T [69], Huang S [70]
Ammonia Exposure	- Sealed chamber: Ammonia concentration 20~50 ppm (upper limit of poultry house standard) - Acute: 80 ppm × 72 h - Chronic: 30 ppm × 4 weeks	Broiler chicken, pig, rat	- Shedding of tracheal cilia - Phagocytic ability of alveolar macrophages ↓	3 days to 8 weeks	Shi Z [71], Wang X [72]
Transport stress	(amplitude 3 cm, frequency 2 Hz)	Stress research in live animal transportation	Accelerated glycogen depletion, CK increases three fold	2 to 12 h	He Y [73], Ma C [74]

↑: Increase in expression level, ↓: Decrease in expression level.

## Data Availability

No datasets were generated or analysed during the current study.

## Abbreviations

The following abbreviations are used in this manuscript:

ACTH, adrenocorticotropic hormone; AP-1, activator protein-1; CAT, Catalase; CRF, corticotropin-releasing factor; COR, corticosterone; DSS, dextran sulfate sodium; GH, growth hormone; GSH, Glutathione; GST, glutathione S-Transferase; GPX, Glutathione peroxidase; HCT, red blood cell specific volume; HPA, hypothalamic–pituitary–adrenal; HPT, hypothalamic–pituitary–thyroid axis; HSP70, heat shock protein 70; IGF-1, insulin-like growth factor; IL-1, interleukin-1; IL-2, interleukin-2; IL-6, interleukin-6; IMN, immunonutrition; INOS, Inducible Nitric Oxide Synthase; LPS, lipopolysaccharide; MDA, malondialdehyde; MPO, myeloperoxidase; NOX2, NADPH oxidase 2; PCO protein carbonyl; PCV, porcine circovirus; PCV-2, Porcine circovirus type 2; PGF2α, Prostaglandin F2α; RNS, reactive nitrogen species; ROS, reactive oxygen species; SAM, sympathetic-adrenal medulla axis; SDA, specific dynamic action; SOD, superoxide dismutase; T-AOC, total antioxidant capacity; TBARS, thiobarbituric acid reactive substances; TNF-α, tumor necrosis factor-α;UCP1, Uncoupling Protein 1; 8-OHdG, 8-hydroxydeoxyguanosine.
